# Stress Reactivity Influences the Relationship between Emotional Labor Strategies and Job Burnouts among Chinese Hospital Nurses

**DOI:** 10.1155/2020/8837024

**Published:** 2020-09-22

**Authors:** Huihua Deng, Hanyao Wu, Xingliang Qi, Caixiang Jin, Jianmei Li

**Affiliations:** ^1^Key Laboratory of Child Development and Learning Science (Southeast University), Ministry of Education, Nanjing 210096, China; ^2^Institute of Child Development and Education, Southeast University, Nanjing 210096, China; ^3^Department of Medical Humanity, School of Humanities, Southeast University, Nanjing 211189, China; ^4^College of Pro-School Education, Nanjing Xiaozhuang University, Nanjing 211171, China; ^5^Department of Nursing, Nanjing Integrated Traditional Chinese and Western Medicine Hospital, Nanjing 210014, China; ^6^Office of Social Science, Southeast University, Nanjing 210096, China

## Abstract

Extant studies mostly focused on the buffering role of social and external organizational resources and personal mental resources. However, there is no research exploring the moderating role of personal physiological resources (e.g., stress reactivity). The present study is aimed at examining the interactive effect of emotional labor and stress reactivity on job burnout. The present study utilized cortisol content in a 1 cm hair segment as the biomarker of total stress reactivity in one month. The participants were 229 female hospital nurses randomly recruited from city hospitals, China. They self-reported their emotional labor strategies and job burnout syndromes and provided 1 cm hair segments closest to the scalp two weeks later after the survey. Hair cortisol content was determined with high-performance liquid chromatography-tandem mass spectrometry. The results revealed that hair cortisol can moderate the associations of surface acting with emotional exhaustion and personal burnout; of deep acting with emotional exhaustion, depersonalization, and personal burnout; and of expression of naturally felt emotions with professional inefficacy. In particular, nurses with high cortisol levels not only showed higher emotional exhaustion than those with low cortisol levels under high surface acting but also showed lower emotional exhaustion under low surface acting. A similar situation was true for nurses' emotional exhaustion and depersonalization in the context of deep acting. Nurses with low hair cortisol levels not only showed higher professional inefficacy than those with high hair cortisol levels under low expression of naturally felt emotions but also showed lower professional inefficacy under high expression of naturally felt emotions. Additionally, nurses with high hair cortisol levels showed lower personal burnout than those with low hair cortisol levels under low surface acting or high deep acting. In summary, the interaction pattern between stress reactivity and emotional labor was varied with the nature of emotional labor strategy and job burnout.

## 1. Introduction

Job burnout is a typical syndrome that results from chronic stress elicited by high job demands [1, 2]. Emotional labor is a particular aspect of job demands, requiring employees to modify their affective displays at work [3]. Intense emotional labor in a long time has been associated with higher job burnout [4] as work tasks with highly physical and other mental demands do [1]. *Nevertheless,* the relationship between emotional labor and job burnout may vary with the nature of emotional labor strategy that is required across numerous occupations and with employees' personal resources. Among various personal resources, stress reactivity conceptualized as a high biological sensitivity to context [5] may be one of the important personal physiological resources improving employees' sensitivity to organizational requirements in emotional labor. However, it is unclear yet whether stress reactivity can buffer the deleterious effect of high emotional labor as personal mental resources buffer the deleterious effect of high job demands, such as self-esteem, self-efficacy, and optimism [6, 7]. Thus, determining the buffering role of stress reactivity will be helpful for understanding more fully the importance of the biological processes in employees dealing with job-related emotional labor. Therefore, the current study is aimed at examining how an emotional labor strategy interacts with employees' stress reactivity in predicting employees' job burnout.

Emotional labor has been conceptualized as a three-dimensional structure separating three strategies: surface acting, deep acting, and the expression of naturally felt emotions [8, 9]. Surface acting refers to employees hiding felt emotions or faking unfelt emotions without shaping inner feelings (acting in bad faith) to fit the desired emotion display at the workplace, and deep acting refers to employees modifying their actual inner emotion states (acting in good faith) while the expression of naturally felt emotions refers to employees spontaneously and genuinely experiencing and displaying the felt emotions [8]. Surface acting entails the consumption of a substantial amount of energy and resources in suppressing the true emotions at the behavioral level. Thus, surface acting perhaps leads to an imbalance between emotional demands and the resource expenditure because employees have strong motivations to conserve and establish their resources to minimize the extent to which they spend resources in their emotional labor at work as suggested by the conservation of resources model [10]. In contrast, deep acting spends very limited resources in the internalization of job demands on one's emotion, and natural feelings are often consistent with the expression normally demanded by the work. Therefore, surface acting is more likely positively associated with burnout symptoms and negative stressful reactions than deep acting and the expression of naturally felt emotion. Indeed, previous empirical studies mostly demonstrated positive associations of surface acting with burnout syndromes (e.g., emotional exhaustion and depersonalization) [11–17] and negative associations of the expression of naturally felt emotion with burnout syndromes [14, 16]. However, the association between deep acting and job burnout was less consistent in the previous, with studies demonstrating negative, positive, or no associations [11, 13, 14, 16–20]. One of the main reasons for the inconsistency might be that the amount and nature of emotional labor demand that employees face varied across numerous occupations requiring employees to perform the form of emotional labor differing in interpersonal interactions, such as mass service and high commitment service [21]. For example, nurses have little chance to establish stable relationships with their massive outpatients during short-term and one-off encounters, thereby having less autonomy to express naturally felt emotions than teachers who can establish relatively stable relationships with the students they well know. Another reason might be that the amount and form of emotional labor strategy employees perform varied with employees' stress reactivity or their sensitivity to organizational requirements in emotional labor. Therefore, it is necessary to separately validate the relationship between each emotional labor strategy and job burnout and examine whether the relationship shows the pattern differing in three emotional labor strategies and whether the relationship is moderated by stress reactivity.

As one of the stress-sensitive nervous systems, the hypothalamic-pituitary-adrenal (HPA) axis is responsible for the cortisol secretion to help organs adapt to stressful events [22]. Naturally, cortisol is considered as a biomarker of the HPA activity. It is also regarded as one of the reliable biomarkers for assessing an individual's stress reactivity [5, 23]. Salivary cortisol levels within one day were ever utilized as a biomarker of stress reactivity [23]. However, salivary or urinary cortisol levels reflect the acute or short-term activity of the HPA axis (or stress reactivity) over several hours or up to one day [24]. These traditional biomarkers do not reliably reflect long-term HPA activity (or stress reactivity) enough to match the time span (e.g., one-month period) that most psychological measurements cover in their questions. Alternatively, hair cortisol has been proven to be a novel biomarker reliably assessing basal cortisol levels and the long-term activity of the HPA axis [25]. That is, if the hair growth rate is 1 cm per month [26], the cortisol content in the 1 cm hair segment would reliably reflect the HPA activity over one month or the total reactivity to all daily stressful events over one month. Moreover, it shows high consistency with the average level of multiple-day salivary cortisols within one month [27, 28]. We therefore used the hair cortisol content as a biomarker of one-month stress reactivity to better match the time span that psychological measurements cover.

Previous empirical studies on this topic were done under the Job Demands-Resources (JDR) model [1] and mostly demonstrated that job resources can buffer the harmful effect of job demands [29, 30]. As health-protecting factors, job sources refer to those physical, psychological, social, or organizational aspects of the job that may be functional in achieving work goals, or reduce job demands at the associated physiological and psychological costs, or stimulate personal growth and development [1]. Moreover, it is emphasized that job resources can be catalogued into external resources (i.e., organizational and social resources) and internal personal resources, such as cognitive features and action patterns [31]. As aspects of the self at the cognitive level, emotional level, and biological level, personal resources that are generally linked to resiliency refer to individuals' sense of their ability to control and impact their environment successfully [32, 33]. Traditionally, regarding the job resources buffering the deleterious effect of job demands, extant studies mostly focused on the external organizational resources related to job characteristics, such as social support, job autonomy, quality of the relationship with the supervisor, and performance feedback [1, 29, 34]. Comparatively, limited studies have examined the buffering role of personal mental resources, such as organizational-based self-esteem, self-efficacy, optimism, compassion satisfaction, and recovery experience as resource replenishment [6, 7, 12, 15, 31, 35, 36]. However, to date, there is little research exploring the moderating role of stress reactivity in the association between job demands (e.g., emotional labor) and job burnout.

Stress reactivity as personal physiological resources might play a moderating role as personal mental resources do. This theoretical hypothesis on stress reactivity possibly obtains additional support from recent empirical studies finding that stress reactivity can moderate the relationship between environmental factors and psychological adaptations [23, 37–40]. It found that compared to those with lower cortisol levels (i.e., lower stress reactivity), adolescents with higher cortisol levels (i.e., higher stress reactivity) not only showed less prosocial behaviors and worse execute functions under more family adversities [23, 37] and more internalizing problems under more stressful events [38, 40] but also showed more prosocial behaviors and better execute functions under less family adversities and less internalizing problems under less stressful events, which was consistent with the differential susceptibility model recently developed by Belsky and collaborators [41, 42]. These results implied that high stress reactivity is a plasticity factor (i.e., it is not only a risk factor under adverse environments but also a promoting factor under supportive environments) or that low stress reactivity is a protective factor under adverse environments. Of course, whether the notions are true in the context of high emotional labor demands is needed to be validated.

Taken together, the present study is aimed at independently examining the interactive effects of three emotional labor strategies and stress reactivity on job burnout under the frame of the JDR model where emotional labor is a particular aspect of job demands and stress reactivity might be the most representative personal physiological resources. The cortisol content in the 1 cm hair segment was utilized as the biomarker of one-month stress reactivity, ensuring that the time span the hair cortisol content reflects matched that the measurements of psychological variables cover. We focused our study on Chinese hospital nurses. This is because the nursing profession is an emotionally demanding occupation. Hospital nurses currently in China utilize emotional labor and control their emotional expressions to meet patients' needs in the increasing demand for quality health care services [43]. In order to make the results more generalized, we examined job burnout syndromes that are measured with compulsory questionnaires, Maslach Burnout Inventory-General Survey (MBIGS) [44] and Copenhagen Burnout Inventory (CBI) [45] where MBI focuses on the long-term consequences under continuous job stress in emotions and interpersonal relationships, such as emotional exhaustion, depersonalization, and professional efficacy, and CBI on the different domains of burnout itself, such as personal burnout, work-related burnout, and client-related burnout. Based on the above background, we expected that each emotional labor strategy and hair cortisol would interact to predict nurses' job burnout syndromes and that high hair cortisol (i.e., high stress reactivity) would be the plasticity factor or that lower hair cortisol (i.e., lower stress reactivity) would be a protective factor in the context of high emotional labor demands.

## 2. Method

### 2.1. Participants

The initial sample consisted of 500 female nurses randomly recruited from nine hospitals in Nanjing City, China. All participants provided written informed consent before inclusion. This study followed the Declaration of Helsinki and was approved by the Health Science Research Ethics Board of Southeast University.

Among them, 456 nurses (91.20%) completed all the questionnaires including demographic information, emotional labor strategy, and job burnouts. 341 out of 456 nurses provided their hair strands and the hair-related information. 112 participants were excluded because they were smokers, alcoholics, or obese (body mass indexes ≥ 30), or with shorter hair (<1 cm) or treated hair (e.g., coloring, perm, or bleached), or had medicine intake (e.g., glucocorticoid and antibiotic drugs) or diseases (e.g., canker sores and inflammation), which might influence the contents of cortisol in hair [46]. Finally, 229 nurses participated in the present study. They worked in different types of working departments: intensive care unit (ICU, 20.96%), emergency intensive care unit (EICU, 8.30%), emergency department (33.62%), radiotherapy department (6.99%), rehabilitation department (16.59%), and others (13.54%) including paediatrics, internal medicine, department of medical psychology, Chinese medicine surgery, neurology, neurosurgery, struma, dental department, orthopaedics, endocrine department, and operating theatre over the past one year. They gave a range of years of working as a nurse, in which 50.22% served less than 5 years, 34.06% served 5-15 years, and 15.72% served over 15 years. Of those, 83.84% nurses were in the 8 h three-shift scheduling and 16.16% nurses in the 12 h two-shift scheduling.

### 2.2. Procedures

After signing the informed consent, participants self-reported with the questionnaires their demographic information including the working department, working duration and shift scheduling, emotional labor strategy, and the status of job burnout over the past one month. In order to match survey data in time span, hair samples (about 20 mg in weight) were collected by a well-trained research assist two weeks later after the questionnaires' collection. This is because 1-3 mm of the hair strands embeds in the skin and the 1-2 mm hair strands closest to the scalp cannot be completely cut with scissors [47] if the hair growth rate is 1 cm per month. As-collected hair samples were sealed with foil to avoid from direct irradiation of the sunlight and then were stored in a dry and dark environment at room temperature until the analysis.

### 2.3. Measures

#### 2.3.1. Emotional Labor Strategy

The 14-item emotional labor strategy scale developed by Diefendorff and his colleagues [8] and translated into Chinese by Bai [48] and Cheung and Tang [49] was used to measure three types of emotional labor strategies: surface acting (7 items), deep acting (4 items), and expression of naturally felt emotions (3 items). These items were slightly modified to make the wording match the job characteristics and context of nurses, for example, “put on an act to deal with patients in an appropriate way” (surface acting), “try to actually experience the emotions that I must show to patients” (deep acting), and “the emotions I express to patients are genuine” (expression of naturally felt emotions). Each item is rated on a 5-point Likert scale ranging from 1 (strongly disagree) to 5 (strongly agree). Higher scores indicate a higher emotional labor strategy. The scale was proven to have good reliability and validity in Chinese workers (e.g., Bai, 2006 for employees in supermarkets, hotels, and hospitals; Cheung and Tang, 2009 for human service professionals; and Yin et al., 2012 for teachers). In the present study, the average score for each subscale was utilized, and Cronbach's alpha coefficient was 0.86, 0.76, and 0.85 for the three subscales.

#### 2.3.2. Job Burnout

Job burnout was measured with Maslach Burnout Inventory-General Survey (MBIGS) developed by Schaufeli et al. [44] and translated into Chinese by Li and Shi [50] and with Copenhagen Burnout Inventory (CBI) developed by Kristensen et al. [45] and translated into Chinese by Yeh et al. [51]. The Chinese version of MBIGS includes 16 items assessing the frequency of nurses experiencing burnout and consists of three subscales measuring emotional exhaustion (5 items), depersonalization (5 items), and professional efficacy (6 items) which we renamed and rated as professional inefficacy for the convenience and consistency with the other five burnout subscales in the results' description. Each item is rated on a 7-point Likert scale ranging from 1 (never) to 7 (always), higher scores indicating heavier burnout. The scale was proven to have good reliability and validity in Chinese workers [50]. In the present study, the average score for each subscale was utilized, and Cronbach's alpha coefficient was 0.94, 0.86, and 0.81 for the three subscales.

The Chinese version of CBI also contains 16 items assessing the degree of physical and psychological fatigue and exhaustion perceived by the nurses in three different aspects, personal burnout (5 items), work-related burnout (5 items), and client-related burnout (6 items). The items in the client-related burnout subscale were slightly modified to make the wording match the job context of nurses. Each item is rated on a 5-point Likert scale ranging from 0 (never) to 4 (always), higher scores indicating heavier burnout. The scale was proven to have good reliability and validity in Chinese workers [51]. In the present study, the average score enlarged 25 times for each CBI subscale was utilized to distinguish MBI and CBI, and Cronbach's alpha coefficient was 0.92, 0.90, and 0.90 for the three subscales.

#### 2.3.3. The Analysis of Hair Cortisol Contents

The detailed procedures of analyzing hair cortisol contents (HCC) were described elsewhere [28]. Briefly, the 1 cm hair strands closest to the scalp were treated by a standard protocol: washing with methanol, cutting into pieces, incubation in methanol, centrifugation, solid-phase extraction, and drying at pure nitrogen gas. The dried residue was redissolved in 50-microliter methanol for cortisol analysis that was done on a Qtrap 3200 liquid chromatography-tandem mass spectrometer (ABI, USA). Cortisol was ionized with atmospheric pressure chemical ionization and identified in the positive ion mode using the multiple reaction monitoring mode. The assay method had good linearity in the range of 0.8-250.0 pg/mg, showing the square coefficient of correlation at 0.99. It also had good sensitivity, accuracy, and precision, showing limits of detection and quantitation at 0.3 and 0.8 pg/mg and intraday and interday coefficients of variation less than 15% and recovery ranging between 85 and 115% [28], which fit the requirements of hair cortisol measurement.

### 2.4. Data Preparation and Analysis Procedures

Prior to analyses, all variables were examined for accuracy of data entry, missing data, data normality, and common-method bias. Data were analyzed by the statistical package SPSS 22.0 for windows. Confirmatory factor analysis was performed by Lisrel 8.70. Percentages of missing data were less than 1.0% for all the predictive and outcome variables, and there was no missing data for the moderating variable. Missing data for all the predictive and outcome variables were handled using the expectation-maximization algorithm [52]. The data distribution normality was examined with a one-sample Shapiro-Wilk test. HCC showed nonnormally distributed (*p* < 0.001) and became normally distributed (*p* = 0.200) after a log transformation that could effectively reduce the skewness and kurtosis. The log-transformed HCC data were used for the next Pearson's correlation analysis and the hierarchical multiple regression analysis. All hierarchical multiple regression analyses were conducted controlling for the nurse's working department, shift pattern, and work duration for nursing.

## 3. Results

### 3.1. Descriptive Statistics

Harman's single-factor test was performed to assess the common method variance biases [53]. An exploratory factor analysis (EFA) (principal components extraction) showed that items on surface acting and each subscale of job burnouts did not generate the unique factor with the explained variance more than 40% for all six subscales of job burnouts. A confirmatory factor analysis (CFA) also demonstrated that the items did not converge on a single factor for all six subscales of job burnouts (RMSEAs > 0.150). Similarly, the other predictors and outcome variables did not generate a single factor. Thus, it was assumed that the common method variance bias was not serious in the present study. The details on EFA and CFA were seen in Tables S1 and S2 in the supplemental materials.

As listed in Table 1, surface acting showed significantly positive correlations with emotional exhaustion, depersonalization, personal burnout, work-related burnout, and client-related burnout (*p*′s < 0.01) but did not correlate with professional inefficacy (*p* > 0.05). Deep acting showed significantly negative correlations with all six aspects of job burnouts (*p*′s < 0.01). Expression of naturally felt emotion also showed significantly negative correlations with all six aspects of job burnouts (*p*′s < 0.05). There were no correlations between emotional labor strategies and hair cortisol content and between hair cortisol content and six aspects of job burnouts (*p*′s > 0.05). Additionally, these variables were not correlated with working duration as a nurse (*p*′s > 0.128). Expression of naturally felt emotion, emotional exhaustion, depersonalization, personal burnout, work-related burnout, and client-related burnout were varied with the working department (*p*′s < 0.05), but it was not true for surface acting, deep acting, HCC, and professional inefficiency (*p*′s > 0.05). Emotional exhaustion, personal burnout, and work-related burnout were varied with shift scheduling patterns (*p*′s < 0.05), but it was not true for emotional labor strategies, HCC, and the other burnouts (*p*′s > 0.246). The details are seen in Table S3 in the supplemental materials.

### 3.2. Multiple Linear Regression Analyses

A total of eighteen 4-step moderated hierarchical regressions were conducted to examine the unique effects of emotional labor strategies and interactive effects between emotional labor strategies and hair cortisol content on six aspects of job burnouts. Prior to the regression analyses, all the predictors except for type variables and moderator were centralized to reduce multicollinearity [54]. Demographic variables (i.e., working department, shift pattern, and working duration as a nurse) were entered into the regression in Step 1 to control for their respective effects. The emotional labor strategy as the predictive variable was separately entered into the equation in Step 2. Hair cortisol content as the moderator variable was entered in Step 3. Lastly, the interaction term between each emotional labor strategy and hair cortisol content was separately entered in Step 4. The amount of an additional explained variance was estimated after each step. The collinearity diagnoses revealed that tolerance was more than 0.2 and the variance inflation factor was less than 5 for all the regression equations, indicating that collinearity was not serious in the present models [55]. Subsequently, the nature and directionality of the significant interactions were investigated using the simple slope analyses proposed by Aiken et al. [54] where the effect of high and low levels (i.e., 1 SD above the mean, *M* + 1 SD, and 1 SD below the mean, *M* − 1 SD) of emotional labor was done in female nurses with high and low levels (i.e., *M* + 1 SD and *M* − 1 SD) of hair cortisol content.

The regression results revealed that surface acting could positively predict emotional exhaustion, depersonalization, personal burnout, work-related burnout, and client-related burnout (*p*′s < 0.01), but it was not true for professional inefficacy (*p* > 0.05) as listed in Tables 2 and 3. Deep acting could negatively predict all six aspects of job burnouts (*p*′s < 0.01) as listed in Tables 4 and 5. Expression of naturally felt emotion could also negatively predict all six aspects of job burnouts (*p*′s < 0.01) as listed in Tables 6 and 7. HCC could not predict any aspects of job burnouts (*p*′s > 0.05). Moreover, the Fisher *Z* test on the regression coefficients revealed that the impact patterns of surface acting on emotional exhaustion, depersonalization, professional inefficacy, personal burnout, work-related burnout, and client-related burnout were distinctly different from those of deep acting and expression of naturally felt emotion (*Z* = 5.611, *Z* = 4.083, *Z* = 5.842, *Z* = 4.202, *Z* = 5.148, and *Z* = 6.417, *p*′s < 0.05 and *Z* = 5.666, *Z* = 2.649, *Z* = 6.465, *Z* = 3.367, *Z* = 5.215, and *Z* = 6.546, *p*′s < 0.05), but there were no differences between deep acting and expression of naturally felt emotion (*Z*′s < 1.96, *p*′s > 0.05). Additionally, surface acting showed the association pattern differing between professional inefficacy and the other two MBIGS dimensions, emotional exhaustion and depersonalization (*Z* = 3.74, *Z* = 3.09, *p*′s < 0.05), and deep acting between professional inefficacy and emotional exhaustion (*Z* = 2.26, *p* < 0.05).

**Figure 1:**
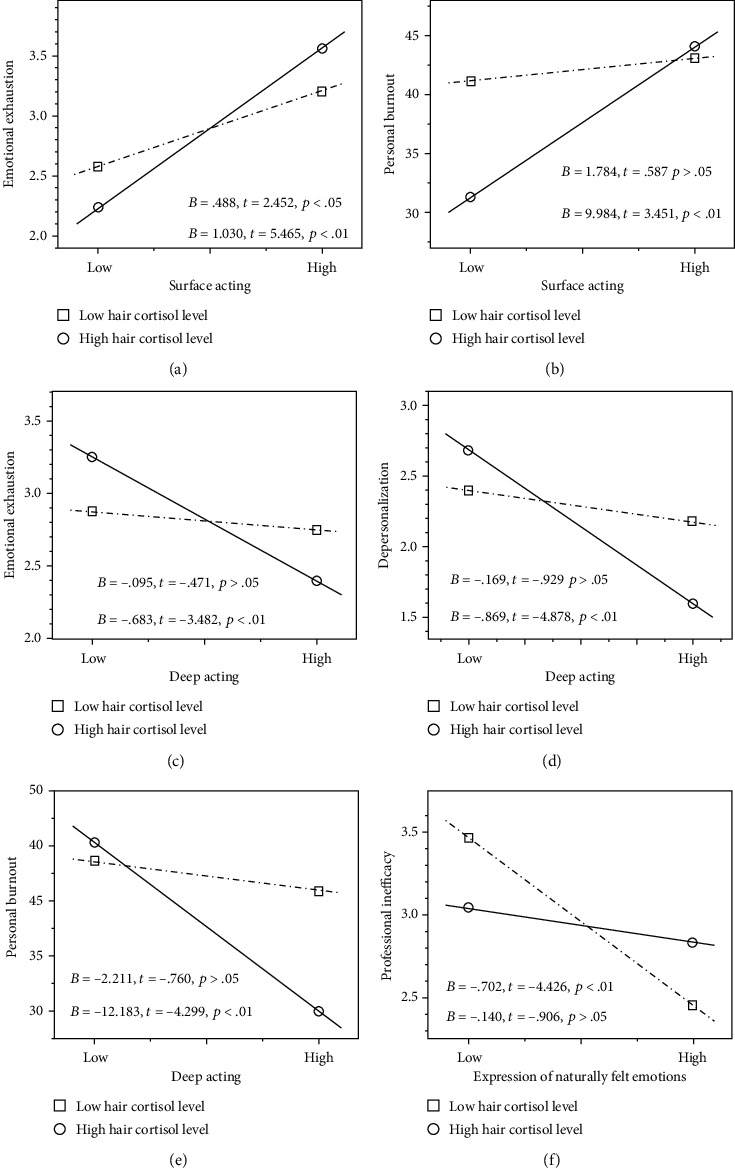
The moderating role of hair cortisol in the relationship between emotional labor strategies and job burnout: (a) surface acting and emotional exhaustion, (b) surface acting and personal burnout, (c) deep acting and emotional exhaustion, (d) deep acting and depersonalization, (e) deep acting and personal burnout, and (f) expression of naturally felt emotions and professional inefficacy.

Notably, the interaction terms between surface acting and HCC could positively predict emotional exhaustion and personal burnout (*p*′s < 0.05) as listed in Tables 2 and 3. The interaction terms between deep acting and HCC could negatively predict emotional exhaustion, depersonalization, and personal burnout (*p*′s < 0.05) as listed in Tables 4 and 5. The interaction terms between expression of naturally felt emotion and HCC could negatively predict professional inefficacy (*p* < 0.05) as listed in Tables 6 and 7. Subsequently, examination of simple slopes revealed that association of emotional labor with job burnout varied across different levels of hair cortisol as shown in Figure 1. A stronger correlation between surface acting and emotional exhaustion was observed in nurses with high cortisol levels (*B* = 1.030, *p* < 0.01) than those with low cortisol levels (*B* = 0.488, *p* < 0.05) although the positive correlation was significant for both nurses with high and low cortisol levels (*p*′s < 0.05). Nurses with high cortisol levels showed higher emotional exhaustion than those with low cortisol levels in the context of high surface acting under which nurses in both groups showed elevated emotional exhaustion but had lower emotional exhaustion in the context of low surface acting (Figure 1(a)). Surface acting was significantly related to higher personal burnout for nurses with high cortisol levels (*B* = 9.984, *p* < 0.01), but it was not true for nurses with low cortisol levels (*B* = 1.784, *p* > 0.05). In comparison to those with low cortisol levels, nurses with high cortisol levels showed lower personal burnout in the context of low surface acting but showed slightly higher personal burnout in the context of high surface acting under which nurses in both groups showed elevated personal burnout (Figure 1(b)).

Deep acting was significantly related to lower emotional exhaustion, depersonalization, and personal burnout for nurses with high cortisol levels (*B* = −0.683, *B* = −0.869, and *B* = −12.183, *p*′s < 0.01), but it was not true for nurses with low cortisol levels (*B* = −0.095, *B* = −0.169, and *B* = −2.211, *p*′s > 0.05). Nurses with high cortisol levels showed higher emotional exhaustion and depersonalization than those with low cortisol levels in the context of low deep acting under which nurses in both groups showed elevated emotional exhaustion and depersonalization but had lower emotional exhaustion and depersonalization in the context of high deep acting (Figures 1(c) and 1(d)). In comparison to those with low cortisol levels, nurses with high cortisol levels had lower personal burnout in the context of high deep acting but showed slightly higher personal burnout in the context of low deep acting under which nurses in both groups showed elevated personal burnout (Figure 1(e)). Expression of naturally felt emotions was significantly related to lower professional inefficacy for nurses with low cortisol levels (*B* = −0.702, *p* < 0.01), but it was not true for nurses with high cortisol levels (*B* = −0.140, *p* > 0.05). Nurses with low cortisol levels showed higher professional inefficacy than those with high cortisol levels in the context of low expression of naturally felt emotions under which nurses in both groups showed elevated professional inefficacy but had lower professional inefficacy in the context of high expression of naturally felt emotions (Figure 1(f)).

## 4. Discussions

The present study found that stress reactivity interacted with emotional labor in predicting emotional exhaustion, depersonalization, professional inefficacy, and personal burnout among Chinese female hospital nurses. Their interaction patterns were varied with the nature of emotional labor strategy and job burnout. Specifically, stress reactivity interacted with surface acting in predicting emotional exhaustion and personal burnout, and with deep acting in predicting emotional exhaustion, depersonalization, and personal burnout, and with the expression of naturally felt emotion in predicting professional inefficacy. The present study further found that low stress reactivity might be a protective factor for Chinese nurses' emotional exhaustion and depersonalization in the context of high job stress due to high surface acting or low deep acting, and high stress reactivity might be a protective factor for Chinese nurses' professional inefficacy in the context of high job stress due to low expression of naturally felt emotions. These findings provided new evidence for the extension of the JDR model to personal physiological resources from social and organizational resources and personal mental resources previous studies had already verified [1, 6, 7, 12, 15, 29, 30, 34–36].

Nurses with high cortisol levels (i.e., high stress reactivity) not only showed higher emotional exhaustion than those with low cortisol levels (i.e., low stress reactivity) when they underwent more stress experience resulting from high surface acting but also showed lower emotional exhaustion when undergoing less stress experience from low surface acting. Similarly, nurses with high stress reactivity not only showed higher emotional exhaustion and depersonalization than those with low cortisol levels under high job stress due to low deep acting but also showed lower emotional exhaustion and depersonalization under low job stress due to high deep acting. Nurses with low stress reactivity not only showed higher professional inefficacy than those with high stress reactivity under high job stress due to low expression of naturally felt emotions but also showed lower professional inefficacy under low job stress due to high expression of naturally felt emotions. These results together with the above-mentioned previous findings on the interaction between stress reactivity and stressful environmental factors in predicting adolescents' psychological adaptations [23, 37, 38, 40] supported the differential susceptibility model. It implied that stress reactivity might be the plasticity factor for Chinese nurses' emotional exhaustion, depersonalization, and professional inefficacy in MBI burnouts. *Nevertheless*, stress reactivity showed the moderating patterns differing between the expression of naturally felt emotions and the other two emotional labor strategies in association with MBI burnout syndromes. For Chinese nurses' emotional exhaustion and depersonalization, high stress reactivity might be the plasticity factor in the context of surface acting and deep acting; that is, high stress reactivity might be a risk factor in the context of high job stress from high surface acting or low deep acting but be a promotive factor in the context of low job stress from low surface acting or high deep acting. In contrast, for Chinese nurses' professional inefficacy, low stress reactivity might be the plasticity factor in the context of the expression of naturally felt emotions. We discuss each of these findings in turn below.

The strengthening effect of high stress reactivity on the association of emotional labor and burnout is possibly attributed to the fact that individuals with higher stress reactivity are more sensitive to the context [5] and are more frequently in the allostasis upon the occurrence of stress [56], thereby being in a relatively higher stress-related arousal state. This might make them more easily influenced by external factors, for example, more easily suffer from adverse environments, thereby undergoing more stress experiences [40, 57]. In the present study, nurses with higher stress reactivity are also more susceptible to the context related to job demands and are more easily affected by job stress, such as stressful emotional labor demands in work environments. This explanation obtains the support from previous findings demonstrating that individuals with high stress reactivity are more likely to show more maladaptive outcomes (e.g., lower prosocial behaviors and more anxiety symptoms) under adverse environments but more adaptive outcomes under supportive environments [23, 37, 38, 40]. However, the explanation is held for associations of surface acting and deep acting with emotional exhaustion and depersonalization, but not for the association between the expression of naturally felt emotions and professional inefficacy. This is perhaps because both surface acting and deep acting need to consume resources to some extent [8]. Surface acting consumes a substantial amount of resources in order to suppress the true emotions at the behavioral level. Deep acting also spends considerable resources in the internalization of job demands on one's emotion through a cognitive regulation and reappraisal although its consumption in resources is relatively limited. In contrast, the expression of naturally felt emotions spends few resources because natural feelings are often consistent with the expression normally demanded by the work. When higher surface acting is exerted, the heavier imbalance between emotional demands and the resource expenditure happens, thereby giving rise to higher stress experience [14] and higher cortisol secretion [58] and more negative outcomes, such as more burnout syndromes as demonstrated in the current study. The resulting major resource's consumption and relatively more burnout syndromes make employees greatly reduce resources to put into improving professional efficacy as suggested by the conservation of resources model [10], thereby weakening the association of surface acting with professional inefficacy as observed in the current study. A similar situation occurs in the context of deep acting that consumes relatively fewer resources than surface acting, but not for the expression of naturally felt emotions. Moreover, because their sensitivity and attention bias to the stressful context, nurses with high stress reactivity would put more physical and cognitive resources to cope with higher stressful emotional labor demands, thereby providing relatively fewer resources to improve their professional efficacy, especially in the context of surface acting and deep acting. On the contrary, because of their insensitivity to job stress, nurses with low stress reactivity would maintain more physical and cognitive resources, thereby having excess resources for improving their professional efficacy, especially in the context of the expression of naturally felt emotions. Additionally, according to the MBI definition of burnout [44], emotional exhaustion, depersonalization, and professional inefficacy are conceptualized as three dimensions that simultaneously occur. Actually, they are independently measured as three distinct and different dimensions as confirmed by many empirical studies [59]. In particular, professional inefficacy is largely independent of the other two dimensions [59]. Emotional exhaustion is seen as an individual state, depersonalization as a coping strategy, and professional inefficacy as one of many consequences of long-term stress as argued in previous studies [45]. Thus, they might have their own characteristic precursors, thereby showing the pattern of associations with emotional labor strategies differing between professional inefficacy and the other two dimensions, such as their association with surface acting as demonstrated in the current study. Therefore, high stress reactivity strengthens the association of surface acting with emotional exhaustion and associations of deep acting with emotional exhaustion and depersonalization and weakens the association of the expression of naturally felt emotions with professional inefficacy as found in the present study. By contrast, low stress reactivity strengthens the association of the expression of naturally felt emotions with professional inefficacy.

Notably, although nurses with high stress reactivity showed lower personal burnout than those with low stress reactivity under low surface acting or high deep acting, there might be no marked difference between the two groups in the context of high surface acting or low deep acting under which both groups showed elevated personal burnout. These results were not consistent with the differential susceptibility model but might be consistent with the vantage sensitivity model positing that individuals with vantage sensitivity are more positively responsive to supportive environments to which they are exposed but are not vulnerable to the negative influence of adverse environments [60]. It implied that for Chinese nurses' personal burnout, high stress reactivity might be a promotive factor in the context of low job stress due to low surface acting and high deep acting while high stress reactivity might be not a risk factor or low stress reactivity might be not a protective factor in the context of high job stress due to high surface acting and low deep acting. Obviously, the interaction of stress reactivity with surface acting and deep acting showed the pattern differing in CBI personal burnout and MBI emotional exhaustion and depersonalization even though both types of syndromes are measured as burnout indices. This might be because the sensitivity to job stress differs between CBI personal burnout and MBI emotional exhaustion and depersonalization. The MBI burnout is defined as a syndrome of emotional exhaustion, depersonalization, and professional inefficacy that can occur among individuals who do the human service work [61]. The core definition remains not changed in BMI-GS where some items were slightly revised to be widely applied across a variety of occupations rather than be restricted to the human service sector [44]. This definition implies that the MBI burnout is directly and primarily caused by the characteristic stress in human service work, the high emotional labor demands. Thus, the MBI burnout has relatively high sensitivity to high emotional labor demands. In the CBI, the core of burnout remains fatigue and exhaustion. However, CBI is designed as a questionnaire with three subdimensions, personal burnout, work-related burnout, and client-related work closely associated with three specific domains in a person's life. Among them, personal burnout is defined to be “the degree of physical and psychological fatigue and exhaustion experienced by the person” [45], and its questions are formulated in a way so that all human beings can answer them. Thus, personal burnout as a truly generic syndrome is relatively insensitive to the high emotional labor demands although there are associations between them. Therefore, high stress reactivity might be a risk factor under high job stress from high surface acting and low deep acting for nurses with higher emotional exhaustion and depersonalization, but not for those with higher personal burnout.

The current study has several limitations. First, the study was a cross-sectional design, limiting the understanding of the directionality on the association between variables. Second, the study was based on a sample of Han Chinese female nurses in city hospitals, China. The generalization of the present findings to other occupations and other ethnic groups in China and non-Chinese populations in other countries with collectivistic cultures and even western countries with individualistic cultures needs to be cautious. Third, the study only utilized a single index, hair cortisol as the biomarker of stress reactivity. Multiple biomarkers of stress reactivity would be recommended in future research. Finally, the study did not consider the influence of other job demands (e.g., work load, mental load, and task complexity) and of socioeconomic status, such as family income, education degree, and nurse's rank although these variables showed strong associations with working duration as a nurse.

In summary, stress reactivity interacted with emotional labor in predicting emotional exhaustion, depersonalization, professional inefficacy, and personal burnout among Chinese hospital nurses. Their interaction patterns were varied with the nature of emotional labor strategy and job burnout. High stress reactivity might be the plasticity factor for Chinese nurses' emotional exhaustion and depersonalization in the context of surface acting and deep acting, and low stress reactivity might be the plasticity factor for Chinese nurses' professional inefficacy in the context of the expression of naturally felt emotions, which supported the differential susceptibility model. Additionally, high stress reactivity might be a promoting factor for Chinese nurses' personal burnout in the context of low surface acting or high deep acting, which might support the vantage sensitivity model.

## Figures and Tables

**Table 1:** Means, standard deviations, and coefficients of correlations for the study variables (*n* = 229).

		1	2	3	4	5	6	7	8	9	10
1 Surface acting	—	—	—	—	—	—	—	—	—	—
2 Deep acting	-0.100	—	—	—	—	—	—	—	—	—
3 Natural expression^a^	-0.425^∗∗^	0.554^∗∗^	—	—	—	—	—	—	—	—
4 HCC^b^	0.067	-0.001	0.036	—	—	—	—	—	—	—
5 Emotional exhaustion	0.383^∗∗^	-0.201^∗∗^	-0.194^∗∗^	0.035	—	—	—	—	—	—
6 Professional inefficacy	0.002	-0.363^∗∗^	-0.246^∗∗^	-0.006	0.108	—	—	—	—	—
7 Depersonalization	0.306^∗∗^	-0.265^∗∗^	-0.311^∗∗^	-0.046	0.654^∗∗^	0.190^∗∗^	—	—	—	—
8 Personal burnout	0.234^∗∗^	-0.253^∗∗^	-0.168^∗^	-0.072	0.658^∗∗^	0.175^∗∗^	0.472^∗∗^	—	—	—
9 Work-related burnout	0.285^∗∗^	-0.265^∗∗^	-0.259^∗∗^	0.013	0.699^∗∗^	0.282^∗∗^	0.566^∗∗^	0.858^∗∗^	—	—
10 Client-related burnout	0.329^∗∗^	-0.330^∗∗^	-0.333^∗∗^	0.010	0.567^∗∗^	0.284^∗∗^	0.562^∗∗^	0.621^∗∗^	0.754^∗∗^	—
*M* ^c^	2.59	3.13	3.44	3.2	3.59	3.00	2.63	52.45	46.63	41.45
SD^c^	0.65	0.63	0.74	0.8-48.7	1.45	1.22	1.32	21.40	20.74	19.28

Notes: ^∗^*p* < 0.05, ^∗∗^*p* < 0.01, and ^∗∗∗^*p* < 0.001. ^a^Natural expression referred to expression of naturally felt emotions. ^b^HCC was log-transformed for Pearson correlation analysis. ^c^HCC was presented as median and range (pg/mg) because HCC has nonnormal distribution, and the other variables were presented as *M* and SD where *M* was mean and SD was standard deviation. Subscales, surface acting, deep acting, and expression of naturally felt emotions in emotional labor, and three subscales, emotional exhaustion, depersonalization, and professional inefficacy, in Maslach Burnout Inventory (MBI) were presented with average scores, but three subscales, personal burnout, work-related burnout, and client-related burnout, in Copenhagen Burnout Inventory (CBI) were presented with 25 times average scores each subscale to distinguish MBI and CBI throughout the text.

**Table 2:** Coefficients of multiple linear regression of surface acting, hair cortisol content, and their interaction against Maslach job burnout (*n* = 229).

	Independent variable	Emotional exhaustion				Professional inefficacy					Depersonalization		
	Predictive variable	Δ*R*^2^	*β*	*B*	SE	Δ*R*^2^	*β*	*B*	SE	Δ*R*^2^	*β*	*B*	SE
Step 1	Demographic variables^a^	0.105^∗∗^				0.015				0.068^∗^			
	ICU		0.268^∗∗^	0.955	0.339		0.073	0.217	0.299		0.232^∗^	0.751	0.315
	EICU		0.228^∗∗^	1.199	0.434		-0.008	-0.037	0.383		0.173^∗^	0.828	0.403
	Emergency		0.364^∗∗^	1.116	0.299		-0.004	-0.010	0.264		0.219^∗^	0.610	0.278
	Radiotherapy		0.065	0.368	0.430		-0.011	-0.053	0.379		-0.076	-0.391	0.400
	Rehabilitation		0.071	0.276	0.339		-0.083	-0.273	0.299		0.047	0.167	0.315
	Shift pattern		0.068	0.269	0.278		0.005	0.016	0.245		-0.045	-0.160	0.258
	Working duration		0.011	0.003	0.015		0.006	0.001	0.013		0.065	0.014	0.014
Step 2	Surface acting	0.110^∗∗^	0.340^∗∗^	0.762	0.137	0.000	-0.002	-0.003	0.129	0.077^∗∗^	0.285^∗∗^	0.581	0.131
Step 3	HCC^b^	0.000	-0.011	-0.045	0.259	0.000	-0.000	-0.001	0.243	0.005	-0.070	-0.271	0.245
Step 4	SA×HCC^c^	0.014^∗^	0.123^∗^	0.801	0.396	0.004	0.063	0.344	0.376	0.000	0.014	0.081	0.379

Notes: ^∗^*p* < 0.05, ^∗∗^*p* < 0.01, and ^∗∗∗^*p* < 0.001. Δ*R*^2^ was the change of *R* square, *β* was standardized regression coefficients, *B* was unstandardized regression coefficients, and SE was standard error of mean (the same below). ^a^Demographic variables include type variables, working department, and shift pattern (i.e., the 8 h three-shift or 12 h two-shift scheduling pattern) and continuous variable (i.e., working duration as a nurse). Because the working department containing six types of departments was a dummy variable, the intensive care unit (ICU), emergency intensive care unit (EICU), emergency department, radiotherapy department, and rehabilitation department were coded as 1 in turn while the other departments as a reference were coded as 0. The 8 h three-shift and 12 h two-shift scheduling patterns were coded as 0 and 1, respectively. ^b^HCC referred to hair cortisol content. ^c^SA×HCC referred to the interaction between surface acting and hair cortisol content.

**Table 3:** Coefficients of multiple linear regression of surface acting, hair cortisol content, and their interaction against Copenhagen job burnout (*n* = 229).

	Independent variable	Personal burnout				Work-related burnout				Client-related burnout			
	Predictive variable	Δ*R*^2^	*β*	*B*	SE	Δ*R*^2^	*β*	*B*	SE	Δ*R*^2^	*β*	*B*	SE
Step 1	Demographic variables^a^	0.120^∗∗^				0.095^∗∗^				0.127^∗∗^			
	ICU		0.227^∗^	11.912	4.955		0.185^∗^	9.391	4.871		0.262^∗∗^	12.377	4.446
	EICU		0.167	12.926	6.350		0.105	7.890	6.241		0.059	4.121	5.697
	Emergency		0.408^∗∗^	18.454	4.373		0.286^∗∗^	12.515	4.298		0.361^∗∗^	14.683	3.923
	Radiotherapy		0.062	5.153	6.295		-0.015	-1.227	6.188		-0.075	-5.647	5.648
	Rehabilitation		0.090	5.141	4.958		0.035	1.971	4.873		0.132	6.837	4.448
	Shift pattern		0.130	7.543	4.067		0.148	8.349	3.998		0.057	2.998	3.649
	Working duration		0.045	0.152	0.220		-0.005	-0.016	0.216		0.132^∗^	0.400	0.198
Step 2	Surface acting	0.028^∗∗^	0.173^∗∗^	5.701	2.111	0.057^∗^	0.245^∗∗^	7.831	2.042	0.018^∗∗^	0.291^∗∗^	8.673	1.834
Step 3	HCC^b^	0.013	-0.118	-7.366	3.944	0.000	-0.018	-1.029	3.844	0.000	-0.011	-0.644	3.453
Step 4	SA×HCC^c^	0.016^∗^	0.127^∗^	12.251	6.040	0.004	0.062	5.821	5.930	0.001	0.035	3.028	5.335

Notes: ^∗^*p* < 0.05, ^∗∗^*p* < 0.01, and ^∗∗∗^*p* < 0.001. ^a^The same coding as Table 2. ^b^HCC referred to hair cortisol content. ^c^SA×HCC referred to the interaction between surface acting and hair cortisol content.

**Table 4:** Coefficients of multiple linear regression of deep acting, hair cortisol content, and their interaction against Maslach job burnout (*n* = 229).

	Independent variable	Emotional exhaustion				Professional inefficacy				Depersonalization			
	Predictive variable	Δ*R*^2^	*β*	*B*	SE	Δ*R*^2^	*β*	*B*	SE	Δ*R*^2^	*β*	*B*	SE
Step 1	Demographic variables^a^												
Step 2	Deep acting	0.028^∗∗^	-0.172^∗∗^	-0.393	0.147	0.130^∗∗^	-0.368^∗∗^	-0.708	0.122	0.061^∗∗^	-0.251^∗∗^	-0.524	0.134
Step 3	HCC^b^	0.000	0.002	0.010	0.272	0.000	-0.002	-0.008	0.227	0.003	-0.060	-0.231	0.247
Step 4	DA×HCC^c^	0.017^∗^	-0.133^∗^	-0.845	0.400	0.001	0.026	0.137	0.337	0.030^∗∗^	-0.174^∗∗^	-1.010	0.362

Notes: ^∗^*p* < 0.05, ^∗∗^*p* < 0.01, and ^∗∗∗^*p* < 0.001. ^a^Demographic variables showed the same results as Table 2. ^b^HCC referred to hair cortisol content. ^c^DA×HCC referred to the interaction between deep acting and hair cortisol contents.

**Table 5:** Coefficients of multiple linear regression of deep acting, hair cortisol content, and their interaction against Copenhagen job burnout (*n* = 229).

	Independent variable	Personal burnout				Work-related burnout				Client-related burnout			
	Predictive variable	Δ*R*^2^	*β*	*B*	SE	Δ*R*^2^	*β*	*B*	SE	Δ*R*^2^	*β*	*B*	SE
Step 1	Demographic variables^a^												
Step 2	Deep acting	0.045^∗∗^	-0.217^∗∗^	-7.313	2.123	0.051^∗∗^	-0.230^∗∗^	-7.530	2.082	0.083^∗∗^	-0.295^∗∗^	-8.959	1.860
Step 3	HCC^b^	0.012	-0.112	-6.988	3.904	0.000	-0.009	-0.562	3.855	0.000	0.001	-0.060	3.445
Step 4	DA×HCC^c^	0.024^∗^	-0.155^∗^	-14.564	5.727	0.011	-0.107	-9.733	5.700	0.001	-0.030	-2.537	5.125

Notes: ^∗^*p* < 0.05, ^∗∗^*p* < 0.01, and ^∗∗∗^*p* < 0.001. ^a^Demographic variables showed the same results as Table 3. ^b^HCC referred to hair cortisol content. ^c^DA×HCC referred to the interaction between deep acting and hair cortisol content.

**Table 6:** Coefficients of multiple linear regression of expression of naturally felt emotions, hair cortisol content, and their interaction against Maslach job burnout (*n* = 229).

	Independent variable	Emotional exhaustion				Professional inefficacy				Depersonalization			
	Predictive variable	Δ*R*^2^	*β*	*B*	SE	Δ*R*^2^	*β*	*B*	SE	Δ*R*^2^	*β*	*B*	SE
Step 1	Demographic variables^a^												
Step 2	Natural expression^b^	0.030^∗∗^	-0.177^∗∗^	-0.347	0.126	0.058^∗∗^	-0.246^∗∗^	-0.406	0.110	0.088^∗∗^	-0.305^∗∗^	-0.544	0.113
Step 3	HCC^c^	0.000	0.007	0.031	0.271	0.000	0.006	0.020	0.236	0.003	-0.051	-0.198	0.244
Step 4	NE×HCC^d^	0.014	-0.122	-0.725	0.377	0.024^∗^	0.159^∗^	0.793	0.326	0.002	-0.042	-0.229	0.341

Notes: ^∗^*p* < 0.05, ^∗∗^*p* < 0.01, and ^∗∗∗^*p* < 0.001. ^a^Demographic variables showed the same results as seen in Table 2. ^b^Natural expression referred to expression of naturally felt emotions. ^c^HCC referred to hair cortisol content. ^d^NE×HCC referred to the interaction between expression of naturally felt emotions and hair cortisol content.

**Table 7:** Coefficients of multiple linear regression of expression of naturally felt emotions, hair cortisol content, and their interaction against Copenhagen job burnout (*n* = 229).

	Independent variable	Personal burnout				Work-related burnout					Client-related burnout		
	Predictive variable	Δ*R*^2^	*β*	*B*	SE	Δ*R*^2^	*β*	*B*	SE	Δ*R*^2^	*β*	*B*	SE
Step 1	Demographic variables^a^												
Step 2	Natural expression^b^	0.019^∗∗^	-0.141^∗∗^	-4.095	1.857	0.053^∗∗^	-0.236^∗∗^	-6.611	1.791	0.089^∗∗^	-0.306^∗∗^	-7.968	1.596
Step 3	HCC^c^	0.011	-0.107	-6.711	3.969	0.000	-0.002	-0.150	0.852	0.000	0.008	0.431	3.433
Step 4	NE×HCC^d^	0.013	-0.115	-10.038	5.513	0.000	-0.019	-1.640	5.389	0.000	0.017	1.310	4.804

Notes: ^∗^*p* < 0.05, ^∗∗^*p* < 0.01, and ^∗∗∗^*p* < 0.001. ^a^Demographic variables showed the same results as seen in Table 3. ^b^Natural expression referred to expression of naturally felt emotions. ^c^HCC referred to hair cortisol content. ^d^NE×HCC referred to the interaction between expression of naturally felt emotions and hair cortisol content.

## Data Availability

The SPSS data used to support the findings of this study are available from the corresponding author upon request.

## Supplementary Materials

Supplementary Materials (1) The common method bias: an exploratory factor analysis (principal components extraction) showed that the emotional labor scale and job burnout scale did not generate the unique factor with the explained variance more than 40% (28.63% for two job burnout scales, 19.31% and 31.77% for MBI and CBI). Similarly, it was true for three subscales of the emotional labor scale and six subscales of job burnout (details seen in Table S1). A confirmatory factor analysis also demonstrated that the emotional labor scale and two job burnout scales did not converge on a single factor (*χ*^2^ = 7251.69, *df* = 989, *χ*^2^*/df* = 7.332, *NFI* = 0.82, *CFI* = 0.85, *GFI* = 0.42, *RMSEA* = 0.17). Similarly, it was true for three subscales of emotional labor and six subscales of job burnout (details seen in Table S2). (2) The influences of demographic variables on the study variables. As listed in Table S3, the variables in this study were not significantly correlated with working duration as a nurse (*p*′s > 0.128). Expression of naturally felt emotion, emotional exhaustion, depersonalization, personal burnout, work-related burnout, and client-related burnout were varied with the working department (F5,223 = 2.363, *p* = 0.041; and F5,223 = 5.028, *p* = 0.001, but it was not true for surface acting, deep acting, HCC, and professional inefficiency *p*′s > 0.05 . Emotional exhaustion, personal burnout, and work-related burnout were varied with shift-work scheduling pattern (F1,227 = 4.232, *p* = 0.041; F1,227 = 6.032, *p* = 0.015; and F1,227 = 7.628, *p* = 0.006, but it was not true for emotional labor strategies, HCC, and the other burnouts (*p*′s > 0.246).
