# Supplementary material for: Stress Reactivity Influences the Relationship between Emotional Labor Strategies and Job Burnouts among Chinese Hospital Nurses
Source: Neural Plast. 2020 Sep 22;2020:8837024. doi: 10.1155/2020/8837024 (PMC7528115; doi:10.1155/2020/8837024)
Supplement: Supplementary Materials — (1) The common method bias: an exploratory factor analysis (principal components extraction) showed that the emotional labor scale and job burnout scale did not generate the unique factor with the explained variance more than 40% (28.63% for two job burnout scales, 19.31% and 31.77% for MBI and CBI). Similarly, it was true for three subscales of the emotional labor scale and six subscales of job burnout (details seen in Table S1). A confirmatory factor analysis also demonstrated that the emotional labor scale and two job burnout scales did not converge on a single factor (χ2 = 7251.69, df = 989, χ2/df = 7.332, NFI = 0.82, CFI = 0.85, GFI = 0.42, RMSEA = 0.17). Similarly, it was true for three subscales of emotional labor and six subscales of job burnout (details seen in Table S2). (2) The influences of demographic variables on the study variables. As listed in Table S3, the variables in this study were not significantly correlated with working duration as a nurse (p′s > 0.128). Expression of naturally felt emotion, emotional exhaustion, depersonalization, personal burnout, work-related burnout, and client-related burnout were varied with the working department (F5,223 = 2.363, p = 0.041; and F5,223 = 5.028, p = 0.001, but it was not true for surface acting, deep acting, HCC, and professional inefficiency p′s > 0.05 . Emotional exhaustion, personal burnout, and work-related burnout were varied with shift-work scheduling pattern (F1,227 = 4.232, p = 0.041; F1,227 = 6.032, p = 0.015; and F1,227 = 7.628, p = 0.006, but it was not true for emotional labor strategies, HCC, and the other burnouts (p′s > 0.246). [file 8837024.f1.docx]

Supplemental materials

1. The common method bias

An exploratory factor analysis (principal components extraction) showed that emotional labor scale and job burnout scale did not generate the unique factor with the explained variance more than 40 % (28.63 % for two job burnout scales, 19.31 % and 31.77 % for MBI and CBI). Similarly, it was true for three subscales of emotional labor scale and six subscales of job burnout (details seen in Table S1).

A confirmatory factor analysis also demonstrated that emotional labor scale and two job burnout scales did not converge on a single factor (*χ*^2^=7251.69, df=989, *χ*^2^/df=7.332, NFI=.82, CFI=.85, GFI=.42, RMSEA=.17). Similarly, it was true for three subscales of emotional labor and six subscales of job burnout (details seen in Table S2).

2. The influences of demographic variables on the study variables

As listed in Table S3, the variables in this study were not significantly correlated with working duration as a nurse (*p*s>.128). Expression of natural felt emotion, emotional exhaustion, depersonalization, personal burnout, worked-related burnout and client-related burnout were varied with working department (*F*_5, 223_=2.363, *p*=0.041; *F*_5, 223_=5.028, *p*<.001; *F*_5, 223_=2.984, *p*=.013; *F*_5, 223_=5.086, *p*<.001; *F*_5, 223_=3.716, *p*=0.003; *F*_5, 223_=5.307, *p*<.001), but it was not true for surface acting, deep acting, HCC and professional inefficiency (*p*s>.05). Emotional exhaustion, personal burnout and worked-related burnout were varied with shift-work scheduling pattern (*F*_1, 227_=4.232, *p*=.041; *F*_1, 227_=6.032, *p*=.015; *F*_1, 227_=7.628, *p*=.006), but it was not true for emotional labor strategies, HCC and the other burnouts (*p*s>.246).

Tables

Table S1 The explained variance (%) on the unique factor between emotional labor and job burnouts as examined with exploratory factor analysis

| Variables | EE ^d^ | PI ^e^ | DP ^f^ | PB ^g^ | WB ^h^ | CB ^i^ |
| --- | --- | --- | --- | --- | --- | --- |
| SA ^a^ | 34.17 | 27.06 | 30.12 | 32.42 | 30.16 | 31.20 |
| DA ^b^ | 39.38 | 36.24 | 33.04 | 39.13 | 37.10 | 39.43 |
| NE ^c^ | 39.81 | 39.66 | 37.42 | 38.13 | 39.14 | 39.18 |
| Emotional labor scale | 21.82 | 19.73 | 20.05 | 20.51 | 20.14 | 20.50 |

Notes: ^a^ SA refers to surface acting. ^b^ DA refers to deep acting. ^c^ NE refers to expression of natural felt emotions. ^d^ EE refers to emotional exhaustion. ^e^ PI refers to professional inefficiency. ^f^ DP refers to depersonalization. ^g^ PB refers to personal burnout. ^h^ WB refers to work-related burnout. ^i^ CB refers to client-related burnout.

Table S2 The results of confirmatory factor analysis on a single factor that contains one emotional labor subscale and one subscale of job burnouts

| Models ^a^ | *χ*^2^ | df | *χ*^2^/df | GFI | CFI | NFI | RMSEA |
| --- | --- | --- | --- | --- | --- | --- | --- |
| SA-EE | 681.452 | 54 | 12.619 | .571 | .651 | .634 | .226 |
| SA-PI | 726.408 | 65 | 11.176 | .592 | .482 | .464 | .211 |
| SA-DP | 895.720 | 54 | 16.587 | .658 | .456 | .445 | .261 |
| SA-PB | 958.037 | 54 | 17.741 | .490 | .440 | .430 | .271 |
| SA-WB | 741.079 | 54 | 13.724 | .550 | .515 | .500 | .236 |
| SA-CB | 693.630 | 65 | 10.671 | .574 | .582 | .561 | .206 |
| DA-EE | 237.507 | 27 | 8.797 | .780 | .830 | .814 | .185 |
| DA-PI | 280.643 | 27 | 10.394 | .747 | .755 | .738 | .203 |
| DA-DP | 233.709 | 35 | 6.677 | .801 | .754 | .726 | .158 |
| DA-PB | 252.792 | 27 | 9.363 | .766 | .793 | .776 | .192 |
| DA-WB | 217.287 | 27 | 8.048 | .793 | .786 | .765 | .176 |
| DA-CB | 227.862 | 35 | 6.510 | .804 | .806 | .781 | .155 |
| NE-EE | 1003.335 | 20 | 50.167 | .408 | .326 | .325 | .464 |
| NE-PI | 778.467 | 20 | 38.923 | .622 | .398 | .396 | .408 |
| NE-DP | 564.627 | 27 | 20.912 | .540 | .470 | .462 | .296 |
| NE-PB | 863.364 | 20 | 43.018 | .441 | .354 | .352 | .429 |
| NE-WB | 657.669 | 20 | 32.883 | .492 | .433 | .429 | .374 |
| NE-CB | 711.336 | 27 | 26.346 | .466 | .438 | .433 | .333 |

Notes: ^a^ SA refers to surface acting. DA refers to deep acting. NE refers to expression of natural felt emotions. EE refers to emotional exhaustion. PI refers to professional inefficiency. DP refers to depersonalization. PB refers to personal burnout. WB refers to work-related burnout. CB refers to client-related burnout.

Table S3 Association of demographic variables with the studied variables (n=229)

| Variables | Working duration ^c^ | Working department ^d^ | Shift schedule ^d^ |
| --- | --- | --- | --- |
| Surface acting | *r*=.033, *p*=.621 | *F*_5,223_=2.249, *p*=.050 | *F*_1,227_=0.578, *p*=.448 |
| Deep acting | *r*=.035, *p*=.595 | *F*_5,223_=1.810, *p*=.112 | *F*_1,227_=0.020, *p*=.888 |
| Natural expression ^a^ | *r*=.021, *p*=.750 | *F*_5,223_=2.363, *p*=.041 | *F*_1,227_=0.019, *p*=.891 |
| HCC ^b^ | *r*=.029, *p*=.657 | *F*_5,223_=1.355, *p*=.243 | *F*_1,227_=0.805, *p*=.371 |
| Emotional exhaustion | *r*=-.035, *p*=.600 | *F*_5, 223_=5.028, *p*<.001 | *F*_1,227_=4.232, *p*=0.041 |
| Professional inefficacy | *r*=-.008, *p*=.908 | *F*_5,223_=0.694, *p*=.629 | *F*_1,227_=0.279, *p*=.598 |
| Depersonalization | *r*=.018, *p*=.783 | *F*_5,223_=2.984, *p*=.013 | *F*_1,227_=0.247, *p*=.619 |
| Personal burnout | *r*=.015, *p*=.823 | *F*_5,223_=5.086, *p*<.001 | *F*_1,227_=6.032, *p*=.015 |
| Work-related burnout | *r*=-.029, *p*=.660 | *F*_5,223_=3.716, *p*=.003 | *F*_1,227_=7.628, *p*=.006 |
| Client-related burnout | *r*=.101, *p*=.128 | *F*_5,223_=5.307, *p*<.001 | *F*_1,227_=1.352, *p*=.246 |

Notes: **p*<.05, ***p*<.01, ****p*<.001. ^a^ Natural expression refers to expression of natural felt emotions. ^b^ HCC was log-transformed for Pearson correlation analysis and variance of analysis. ^c^ Association between working duration as a nurse and the studied variables was tested with Pearson correlation analysis. ^d^ Association of working department and shift schedule with the studied variables was examined with variance of analysis.
